# Current Status, Clinical Outcomes and Socioeconomic Challenges of Uniportal Video-Assisted Thoracoscopic Surgery in Japan Compared with Robot-Assisted Thoracic Surgery: A Narrative Review

**DOI:** 10.3390/cancers18203318

**Published:** 2026-10-14

**Authors:** Takahiro Homma, Shota Awane, Moeka Tamura, Norifumi Kakizaki, Takayuki Hatakeyama, Kanji Otsubo, Hiroki Sakai, Hideki Marushima, Koji Kojima, Hisashi Saji

**Affiliations:** Department of Thoracic Surgery, St. Marianna University School of Medicine, Kawasaki 216-8511, Japan; shota.awane@marianna-u.ac.jp (S.A.); moeka.tamura@marianna-u.ac.jp (M.T.); norifumi.kakizaki@marianna-u.ac.jp (N.K.); takayuki.hatakeyama@marianna-u.ac.jp (T.H.); kanji.otsubo@marianna-u.ac.jp (K.O.); h2sakai@marianna-u.ac.jp (H.S.); kitamarushi@gmail.com (H.M.); k2kojima@marianna-u.ac.jp (K.K.); saji-q@ya2.so-net.ne.jp (H.S.)

**Keywords:** uniportal, video-assisted thoracoscopic surgery, robot-assisted thoracic surgery, minimally invasive surgery

## Abstract

Multiportal video-assisted thoracoscopic surgery (VATS) was introduced in Japan in the 1990s, with uniportal VATS (U-VATS) and robot-assisted thoracic surgery (RATS) following in the 2010s. U-VATS gained significant traction after the 1st Fellowship of the Japanese Association for Chest Surgery (JACS; currently the Japanese Society for Thoracic Surgery, JSTS) in 2018, leading to the establishment of the Japanese Uniportal VATS Interest Group (JUVIG) in April 2018. JUVIG has since driven wider adoption through extensive activities, including educational programs, publications, and collaborative multicenter studies. As a result, expert surgeons now utilize U-VATS for complex procedures such as bronchoplasty, completion lobectomy, complex segmentectomy, and pediatric surgery. Concurrently, RATS gained insurance coverage in Japan in 2018 and has become more prevalent than U-VATS. Despite the significant costs associated with RATS, its adoption rate surpasses that of U-VATS due to institutional policies, perceived ease of implementation, branding, recruitment incentives, and recent national healthcare insurance reimbursement revisions (add-ons) introduced in 2026, which may create administrative pressure to prioritize robotic cases. Although reduced-port RATS is gaining popularity—blurring the lines with U-VATS—and newly emerging indigenous robotic platforms are expected to further accelerate RATS adoption, multiportal VATS (M-VATS) remains the dominant approach in many institutions. Furthermore, maintaining proficiency in VATS, U-VATS, and open thoracotomy remains indispensable for surgical education, economic viability, and acute intraoperative crisis management. Ultimately, while U-VATS represents a promising minimally invasive option, its widespread adoption for major and complex resections in Japan continues to be restricted by both educational and societal barriers.

## Simple Summary

Multiportal video-assisted thoracoscopic surgery (M-VATS) was introduced in Japan in the 1990s, followed by uniportal VATS (U-VATS) and robot-assisted thoracic surgery (RATS) in the 2010s. U-VATS gained significant traction after the 1st Fellowship of the Japanese Association for Chest Surgery (JACS; currently the Japanese Society for Thoracic Surgery, JSTS), leading to the establishment of the Japanese Uniportal VATS Interest Group (JUVIG). JUVIG has since driven wider adoption through extensive educational programs and collaborative studies, enabling experts to utilize U-VATS for complex resections such as bronchoplasty and complex segmentectomy. Concurrently, RATS obtained insurance coverage in 2018 and has grown more prevalent than U-VATS, influenced by institutional branding, recruitment incentives, and evolving healthcare reimbursement structures. Although newly emerging robotic platforms continue to accelerate RATS adoption, maintaining proficiency in VATS and open thoracotomy remains indispensable for comprehensive surgical education, economic sustainability, and intraoperative crisis management.

## 1. Introduction

The development of thoracoscopy marked a definitive shift toward minimally invasive surgery (MIS), moving the field from open thoracotomy to video-assisted thoracic surgery (VATS). Initially, VATS was performed via mini-thoracotomy; it subsequently evolved to complete multiportal VATS (M-VATS), the then-dominant approach. Following this, uniportal VATS (U-VATS) was pioneered by Dr. Diego González-Rivas [1] and introduced in Japan in the 2010s [2,3]. Its adoption accelerated following U-VATS international training courses and the 1st Fellowship of the Japanese Association for Chest Surgery (JACS; currently the Japanese Society for Thoracic Surgery, JSTS) in 2018, serving as a key turning point for domestic academic recognition [4]. Despite the COVID-19 pandemic, the Japanese Uniportal VATS Interest Group (JUVIG) sustained U-VATS promotion through educational materials, seminars, publications, and device development [2,5].

Initial skepticism from M-VATS proponents gradually diminished as comparative studies demonstrated that U-VATS offers perioperative outcomes and safety profiles comparable to M-VATS, with potential benefits in reducing postoperative pain, while early Japanese clinical series confirmed its technical feasibility across various anatomical resections [5,6,7,8,9,10,11].

Accordingly, this narrative review aims to synthesize current evidence comparing U-VATS and RATS in Japan, evaluating both clinical outcomes—including perioperative metrics, postoperative pain, oncological radicality, learning curves, and cost-effectiveness—and the socioeconomic, educational, and reimbursement factors that shape their real-world institutional adoption.

## 2. Methods

To synthesize the current evidence and identify key differences between surgical approaches, a comprehensive literature search was conducted using the PubMed database in September 2026. Search terms included combinations of the following keywords: ‘thoracic surgery’, ‘video-assisted thoracoscopic surgery’, ‘thoracotomy’, ‘multiport’, ‘multiportal’, ‘uniport’, ‘uniportal’, ‘single incision’, ‘single port’, ‘robot’, ‘robot-assisted’, ‘learning curve’, ‘morbidity’, ‘mortality’, ‘complication’, ‘pain’, ‘lymph node dissection’, ‘lymphadenectomy’, ‘conversion’, ‘training’, ‘surgical education’, ‘cost’, and ‘surgical technique’. Original articles, proceedings papers, and review articles published up to the search date containing these terms in their titles or abstracts were screened. Additionally, reference lists of the identified publications were manually reviewed to capture further relevant studies, resulting in a total of 75 papers included in this review. The retrieved information was synthesized using a narrative approach and categorized into five main domains corresponding to the core sections of this manuscript: (1) RATS Dominance and Discrepancy; (2) Socioeconomic Drivers and Healthcare Insurance System; (3) Educational Ecosystem, Systemic Constraints, and Safety Considerations; (4) Evaluation Systems and Institutional Barriers; and (5) Global Perspectives and Future Outlook.

## 3. Bibliometric Analysis and Author Identification

A bibliometric analysis was conducted using the Dimensions database (Digital Science; https://www.dimensions.ai (accessed on 1 October 2026)) on 15 August 2026 to identify active research hubs across the four surgical approaches (RATS, open thoracotomy, U-VATS, and M-VATS). Standardized search strategies were applied for each surgical approach using specific keyword combinations [e.g., (“uniportal” OR “single-port”) AND (“VATS” OR “video-assisted”) AND “lung”].

For each approach, the top 50 authors were ranked based strictly on publication volume. Author disambiguation was performed utilizing unique Dimensions Researcher IDs, cross-referenced with institutional affiliations, ORCID identifiers, and publication histories to minimize misclassification from identical names or split profiles. Authors who met the top 50 threshold in more than one surgical category were included independently in each respective category’s author pool to reflect approach-specific productivity.

## 4. RATS Dominance and Discrepancy

Robot-assisted thoracic surgery (RATS), also introduced in the 2010s, is currently more prevalent in Japan, with an institutional adoption rate of 57.5% vs. 42.5% for U-VATS [12]. RATS faces persistent challenges, including high capital costs, a multiportal configuration, and ongoing limitations in insurance coverage and system availability [13,14]. While U-VATS is arguably less invasive based on wound size and pain, RATS has achieved broader institutional adoption [12].

**Figure 1:**
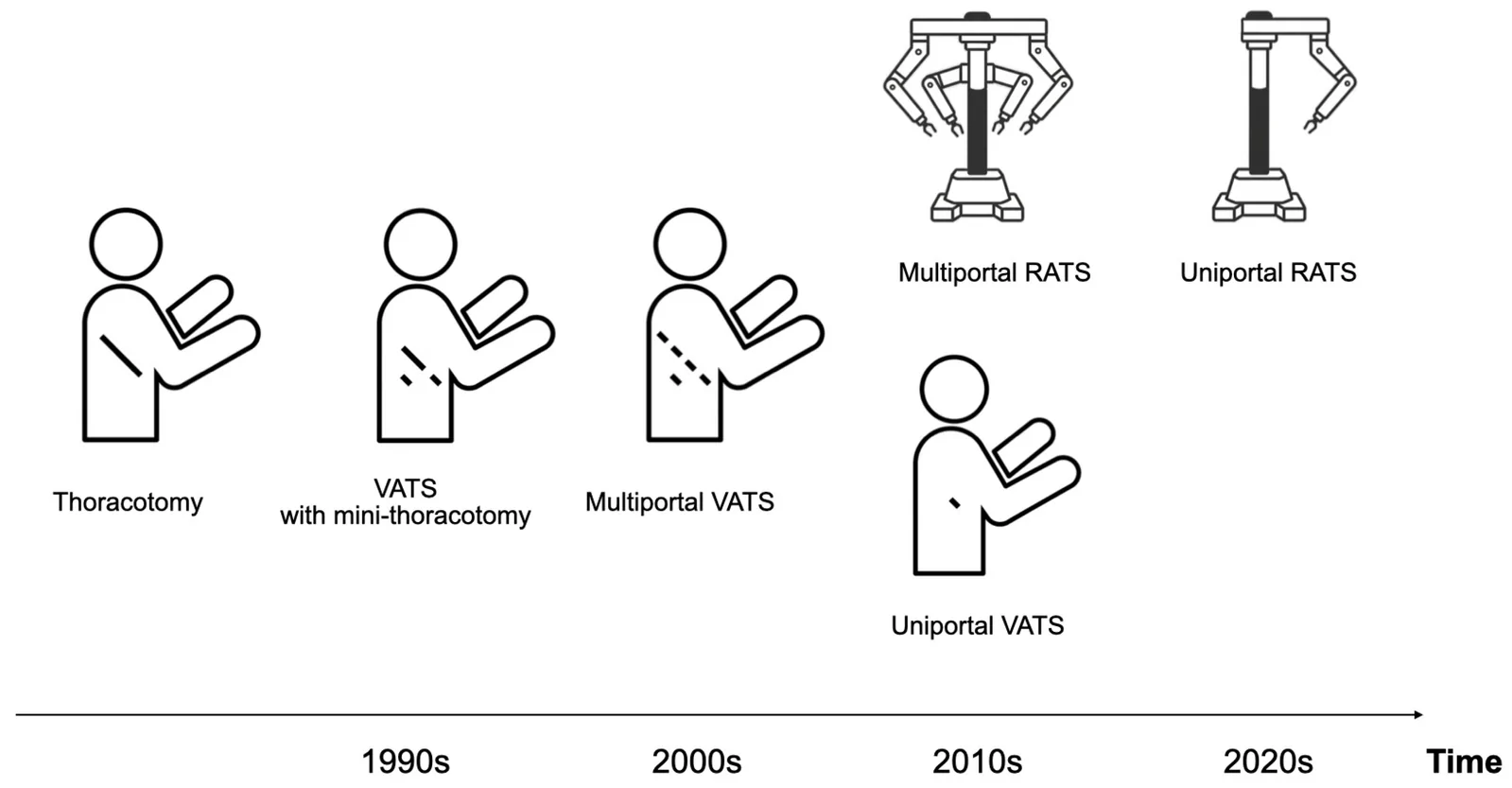
The evolution of minimally invasive thoracic surgery: parallel trends toward uniportal convergence. The evolution of minimally invasive thoracic surgery has proceeded along two distinct tracks: port reduction via video-assisted thoracoscopic surgery (VATS) and enhanced maneuverability via robot-assisted thoracic surgery (RATS). Both streams are currently converging toward the ultimate uniport approach.

This observed discrepancy is suggested to be influenced by a complex interplay of several factors: (1) institutional economic motivations to optimize the operational utilization of capital-intensive robotic platforms, (2) the perceived technological advancement and competitive institutional positioning—which may facilitate patient engagement and support the recruitment of young surgical trainees amidst Japan’s severe shortage in the surgical workforce [15,16]—and (3) the technical complexity of U-VATS combined with a relative scarcity of qualified instructors and mentorship programs. Consequently, the clinical adoption of these surgical modalities is shaped not only by technical feasibility but also by institutional healthcare economics, organizational strategies, and educational resources. Meanwhile, RATS itself is evolving from traditional multiportal approaches toward reduced-port and uniportal platforms [17,18,19] (Figure 1).

To provide a balanced synthesis of both modalities, a comprehensive comparison between U-VATS and RATS across clinical, technical, educational, and economic domains is summarized in Table 1.

## 5. Perioperative Outcomes

(1) U-VATS vs. M-VATS

Compared to M-VATS, U-VATS significantly reduces intraoperative blood loss (1 mL vs. 30 mL) and shortens hospital length of stay (5 days vs. 8 days). Shorter incision and closure times contributed to reduced overall operative durations (141 min vs. 183 min), alongside lower overall complication rates (23.9% vs. 40.9%). Because U-VATS utilizes a single incision site, it lowers the incidence and shortens the duration of postoperative neuropathic pain (30 days vs. 60 days), making epidural-free pain management safe and feasible [5].

Comprehensive meta-analyses comparing U-VATS and M-VATS demonstrated that U-VATS achieves equivalent intraoperative safety and procedural efficacy while offering significant recovery advantages [20,21,22,23,24,25]. Regarding intraoperative metrics, pooled quantitative analyses reveal no statistically significant differences between U-VATS and M-VATS in operative duration, intraoperative blood loss, conversion rates to open thoracotomy or additional ports, or the total number of resected lymph nodes and stations. Conversely, U-VATS delivers clear advantages in postoperative recovery, including a significantly lower overall complication rate, shortened chest tube drainage duration, reduced postoperative hospital length of stay, and lower visual analog scale (VAS) pain scores in the early recovery phase. Overall, these systematic reviews confirm that U-VATS provides comparable operative safety and oncological radicality to M-VATS without compromising procedural feasibility, while enhancing minimally invasive patient recovery through reduced surgical trauma.

Furthermore, recent nationwide real-world registry data from Japan (N = 3546) confirm the safety and feasibility of U-VATS in routine practice, demonstrating an exceptionally low 30-day mortality rate of 0.3% and low conversion rates to multiport VATS (1.8–2.6%) or open thoracotomy (2.4–3.6%) for both lobectomy and segmentectomy. In a propensity-score-weighted comparison (IPTW, N = 2530), U-VATS segmentectomy achieved surgical procedure-related complication rates comparable to lobectomy (OR: 0.89), while offering significantly shorter chest drain duration, shorter hospital stay, lower Clavien–Dindo grade ≥ III morbidity (7.1% vs. 10.0%; OR: 0.69), and fewer 30-day readmissions (0.5% vs. 2.4%; OR: 0.19). Additionally, institutional surgical volume significantly impacts technical safety: high-volume centers (≥128 cases) exhibit a 46% reduction in intraoperative significant vessel injuries compared with low-volume centers (OR: 0.54, *p* = 0.011) and a 37% reduction in postoperative prolonged air leaks compared with middle-volume centers (OR: 0.63, *p* = 0.006), underscoring the importance of accumulated team expertise in optimizing U-VATS outcomes [9,10,11].

(2) RATS vs. M-VATS/U-VATS

No clinically significant differences were observed between RATS and VATS (including U-VATS) regarding overall complication rates (RATS 13.7% vs. VATS 15.1%), major complication rates, 30-day mortality, or hospital stay, demonstrating comparable safety and efficacy in routine lung resections [26]. However, RATS achieved significantly superior results in lymph node yield (17.6 for RATS vs. 13.7 for U-VATS) [27,28] and demonstrates clear technical advantages in highly complex procedures—such as bronchoplasty and sleeve resections—owing to its multi-articulated instrumentation and high-definition 3D visualization. Due to the required system setup and robotic arm docking, operative times tend to be longer for RATS than for VATS [29,30].

Regarding conversion dynamics, large-scale STS database analysis (N = 27,695) indicates that while overall conversion to open thoracotomy is lower in RATS than in VATS (6.0% vs. 11.0%, *p* < 0.001), open conversions during RATS occur emergently (17.9% vs. 9.6%, *p* < 0.001) and due to vascular injury (26.8% vs. 14.3%, *p* < 0.001) significantly more frequently than in VATS [31]. Consequently, RATS conversions carry higher odds of requiring intraoperative blood transfusions (OR: 1.68, *p* = 0.024) and a higher median volume of transfused units (3.0 vs. 2.0 units, *p* = 0.0096). Nevertheless, overall mortality after open conversion remains comparable between approaches (3.1% for RATS vs. 2.2% for VATS, *p* = 0.24). Conversely, nationwide U-VATS data showed that even when intraoperative significant vascular injury occurs (3.4–3.5%), approximately 60% to 70% of these events can be successfully managed thoracoscopically without open conversion, highlighting the advantage of immediate bedside intervention in uniportal surgery.

## 6. Postoperative Pain

(1) U-VATS vs. M-VATS

Compared to M-VATS, U-VATS demonstrated distinct pain-reducing benefits. Although no significant difference is observed in acute pain during the early postoperative period (up to POD 5), U-VATS significantly lowers the incidence of postoperative neuropathic pain (32.4% vs. 52.1%) and substantially shortens its duration (30 days vs. 60 days). This is attributed to having a single incision site affecting only one intercostal space and utilizing a soft wound retractor rather than rigid trocar ports, thereby minimizing direct physical compression on the intercostal nerve. Owing to the minimal incision trauma and reduced operative duration of U-VATS, effective pain control equivalent to regimens incorporating epidural anesthesia can be achieved without epidural catheter placement. Consequently, this allows patients to avoid epidural-related adverse events—such as nausea, vomiting, lower-limb motor paralysis, and urinary retention—which occur in approximately 42% of cases managed with epidural anesthesia [5].

Systematic reviews and meta-analyses evaluating postoperative pain between U-VATS and M-VATS consistently demonstrate that U-VATS provides clinically meaningful reductions in acute postoperative pain and analgesic requirements [20,21,23,24,32]. In the most comprehensive meta-analysis to date involving 19 studies and 2544 patients, U-VATS significantly reduced pain intensity at 24 h postoperatively (standardized mean difference [SMD]: −0.98, 95% CI: −1.12 to −0.84, *p* < 0.0001), representing a 2.5-point decrease on a 10-point scale that exceeds the minimal clinically important difference. This pain-reducing benefit persisted through 48 h (SMD: −0.80) and 7 days (SMD: −0.58) postoperatively, accompanied by an 11.5% reduction in postoperative opioid consumption (mean difference: −10.6 mg morphine equivalents). Similarly, propensity score-matched meta-analyses for anatomical lung resections and segmentectomies confirm significantly lower Visual Analog Scale (VAS) pain scores with U-VATS on POD1, POD3, and POD7 compared to M-VATS.

(2) RATS vs. M-VATS/U-VATS

*Short-term to recovery phase comparison*

A prospective comparative study conducted across 12 centers in Japan (RATS: n = 196 vs. VATS: n = 209, with approximately half of the VATS cohort undergoing U-VATS) reported the following outcomes [33]. At 30 days postoperatively, the proportion of patients reporting mild or minimal pain (Numeric Rating Scale [NRS] ≤ 3) was 82.0% in the RATS group compared with 94.7% in the VATS group [4]. Consequently, non-inferiority of RATS relative to VATS was not demonstrated, indicating significantly better pain control in the VATS group. Mean NRS scores and painDETECT scores (a validated metric for neuropathic pain) at postoperative day (POD) 10 and POD 30 were both significantly higher in the RATS group than in the VATS group, accompanied by a higher trend in overall analgesic medication consumption in the RATS group.

Conversely, a large retrospective cohort study by Kwon et al. [34]. (n = 502) demonstrated no statistically significant differences in daily acute pain trajectories (POD 1–9, *p* = 0.647) or daily morphine consumption between RATS and VATS, though both minimally invasive approaches yielded significantly less acute pain than open thoracotomy.

*Long-term and chronic pain comparison (6 months to 2 years)*

Long-term postoperative pain outcomes appear equivalent between RATS and VATS across multiple international cohorts. Studies evaluating chronic pain between 6 months and 2 years postoperatively observed no significant differences in the proportion of pain-free patients (VATS 62.7% vs. RATS 51.2%) or overall chronic pain prevalence (RATS 34.6% vs. VATS 19.8%, *p* = 0.183) [35]. Furthermore, Lacroix et al. confirmed that post-robotic lobectomy pain was predominantly non-neuropathic and caused minimal interference with daily activities [36]. Intriguingly, Kwon et al. identified a notable psychological divergence between objective pain scores and subjective patient perception: significantly more RATS patients (69.2%) believed their surgical approach positively impacted their pain compared to VATS patients (44.2%, *p* = 0.033), highlighting a potential expectation despite equivalent clinical pain metrics [34].

(3) Key risk factors influencing postoperative pain

Postoperative pain dynamics are governed by three primary domains:

1. Port Count and Intercostal Access:

The extent of intercostal trauma is the primary surgical determinant of pain. Multivariable analysis confirms that each single-port reduction decreases the risk of moderate-to-severe pain at POD 30 by 43.6% (OR: 0.564, *p* < 0.001) [33]. By restricting surgical manipulation to a single space, U-VATS significantly reduces neuropathic pain incidence (32.4% vs. 52.1%, *p* = 0.027) and median pain duration (30 vs. 60 days, *p* = 0.041) compared to M-VATS [5].

2. Operative Time and Procedural Complexity:

Prolonged operative duration (≥2.5 h) and thoracotomy/conversion are independent risk factors for neuropathic pain [37]. In challenging cases requiring prolonged operative time (≥5 h) or emergency conversion, the incidence of neuropathic pain increases significantly to 71.4% (vs. 22.0% in suitable VATS, *p* < 0.0001) [38].

3. Patient-Specific and Clinical Predictors:

Independent multivariable predictors of acute pain and neuropathic pain include younger age (*p* < 0.0001), female sex (*p* = 0.036), preoperative baseline narcotic (*p* = 0.014) or hypnotic use, and higher daily opioid consumption (*p* = 0.003) [38,39]. Furthermore, major postoperative complications independently predict chronic numbness (*p* = 0.0003) [34].

In summary, minimizing intercostal access points (via U-VATS or reduced-port RATS) and controlling operative duration, combined with targeted pain management for high-risk patient subgroups, represent the key technical and clinical strategies for thoracic pain mitigation.

## 7. Oncological Outcomes

(1) Overall Conclusion

Evidence from prospective randomized controlled trials (RCTs), large-scale meta-analyses, and propensity score-matched (PSM) analyses consistently demonstrates no statistically significant differences in overall survival (OS), disease-free survival (DFS)/progression-free survival (PFS), or local recurrence rates between RATS and U-VATS (as well as M-VATS), proving oncological equivalency and non-inferiority between the two modalities.

(2) Comparison of Survival Rates (OS and DFS/PFS)

Prospective Randomized Controlled Trial: The three-year OS [40] was 94.6% for RATS vs. 91.5% for VATS (HR: 0.65, 95% CI: 0.33–1.28, *p* = 0.21), satisfying the predefined non-inferiority margin (−5%) and confirming non-inferiority. The five-year OS was 90.7% for RATS vs. 86.3% for VATS. The three-year DFS was 88.7% for RATS vs. 85.4% for VATS (HR: 0.87, 95% CI: 0.50–1.52, *p* = 0.62). The five-year DFS was 83.7% for RATS vs. 82.5% for VATS [40].

Reconstructed Individual Patient Data Meta-Analysis [41]: An analysis of 10 studies (n = 2045) with a median follow-up of 51.7 months demonstrated equivalent long-term OS (HR: 1.05, *p* = 0.538) and DFS (HR: 1.07, *p* = 0.371), with no statistically significant differences.

Direct Comparison of RATS vs. U-VATS [27]: In anatomical lobectomy for early-stage non-small cell lung cancer (NSCLC, Stage I–IIA; n = 231), no significant differences were observed between groups in 1-year DFS (*p* = 0.788) or 1-year OS (*p* = 0.805).

Long-Term Outcomes of U-VATS Segmentectomy [39]: In a 1600-patient PSM analysis comparing U-VATS and 3-port VATS segmentectomy, 5-year OS (97.7% vs. 99.4%, *p* = 0.78) and 5-year PFS (99.7% vs. 98.2%, *p* = 0.18) were equivalent, with comparable local recurrence (0.5% vs. 0.41%) and distant metastasis rates.

Current evidence suggests broadly comparable oncological outcomes between RATS and VATS approaches; however, high-level direct comparative evidence specifically between RATS and U-VATS remains limited.

(3) Lymph Node Dissection and Pathological Staging

Clear approach-specific technical characteristics are observed regarding the extent and precision of lymphadenectomy:

Superiority in dissected count and stations [27,42]: Attributable to high-definition 3D visualization and multi-articulated instruments with 7 degrees of freedom, RATS yields a significantly higher number of harvested lymph nodes (15.0 vs. 10.0, *p* < 0.001) and mediastinal lymph node stations (6.0 vs. 4.0, *p* < 0.001) compared with U-VATS. For hilar and intralobar lymph node dissection (N1 level, Station 12, etc.), RATS enables more precise harvesting, significantly increasing the number of retrieved stations (5.86 vs. 5.16, *p* = 0.014).

Impact on long-term prognosis [27,42]: Although the higher lymph node yield in RATS may contribute to more accurate pathological N staging, current clinical evidence indicates that this does not translate into a statistically significant improvement in long-term OS or DFS. Clinically, both approaches achieve adequate and thorough oncological radicality.

## 8. Cost-Effectiveness & Financial Viability

Direct intraoperative costs for RATS are substantially higher than those for open thoracotomy or VATS due to system depreciation expenses and high consumable costs, including specialized wristed instruments and sterile drapes.

Conversely, minimally invasive approaches (both RATS and VATS) reduce hospital length of stay and post-surgical complication risks compared to open thoracotomy. While some health economics models suggest that savings in postoperative management and nursing care may offset initial intraoperative expenditures within total direct hospitalization costs, conclusive evidence demonstrating that RATS provides sufficient financial offset to fully recover its capital investment remains limited.

## 9. Socioeconomic Drivers and Healthcare Insurance System

Under Japan’s National Health Insurance (NHI) system, the adoption of RATS has been significantly influenced by evolving reimbursement policies. Historically, substantial capital outlay and ongoing consumable expenditures rendered RATS less financially favorable for institutions compared to conventional VATS or U-VATS [13,14], prompting health facilities to utilize robotic systems in institutional marketing and capital equipment amortization. This landscape has further evolved with recent NHI reimbursement revisions, which introduced dedicated financial add-on fees (kasan) for RATS [43]. While these reimbursement adjustments aim to ensure baseline institutional viability, they may also potentially influence administrative priorities regarding robotic case volumes. Consequently, surgical approach selection can be influenced by institutional financial structures alongside purely clinical considerations. Nevertheless, NHI coverage and these financial add-on fees remain restricted to specific malignant indications and designated surgical procedures.

From the perspectives of health economics and surgical management, the revised reimbursement structure can be conceptualized as potentially altering surgical decision-making across four primary dimensions:

(1) Volume Thresholds & Hospital Profitability:

Revisions to facility standards, such as the newly introduced add-on bonus for robotic-assisted surgery requiring ≥ 200 annual cases for specific designated procedures, create a strong financial incentive for mid-to-large-scale hospitals operating near this volume boundary [43]. To cross the threshold and maximize institutional profitability, administrators and clinical leaders may create strong financial incentives for administrators and clinical leaders to expand robotic indications and centralize surgical volume [44,45].

(2) Procedure-Specific Margin Differences:

Disparities in reimbursement points—where procedures supported by robust clinical superiority evidence receive higher add-on tariffs while others do not—directly alter the profit margins of individual operations. This financial gradient may shift the clinical threshold when choosing between open surgery, conventional minimally invasive techniques (e.g., VATS or standard laparoscopy), and robotic-assisted approaches [46].

(3) Multidisciplinary Platform Sharing:

In the allocation of limited operating room block schedules among multiple surgical departments (e.g., Urology, Gastrointestinal Surgery, Thoracic Surgery, and Gynecology), robotic system access is strategically prioritized. Consequently, departments generating higher reimbursement margins or contributing significantly to the hospital’s annual volume threshold may potentially be favored in resource allocation, obtaining greater access to the robotic platform [47,48,49].

(4) Physician Practice Patterns & Board Certification:

Institution-level facility criteria set by health authorities operate in tandem with surgical society credentialing systems (such as proctorship programs and institutional certifications). These dual requirements directly shape individual surgeons’ practice patterns, defining clinical indications and guiding patient case selection [50,51].

This ongoing shift underscores an important policy consideration: in the absence of definitive clinical superiority of RATS over U-VATS across all routine indications, aligning institutional economic incentives with patient-centered, individualized surgical decision-making remains a pivotal challenge for healthcare leadership [52,53].

## 10. Educational Ecosystem, Systemic Constraints, and Safety Considerations

These advancements reflect Japan’s decade-long MIS trend, yet conservative institutions still predominantly rely on M-VATS [12]. This reliance presents a striking paradox given that some facilities aggressively adopt RATS primarily to recruit young surgeons [15,16]; despite this recruitment-driven push for advanced robotics, senior surgeons often lack the expertise to teach U-VATS. A perception persists that M-VATS proficiency is necessary before advancing to U-VATS or RATS. This mindset closely mirrors the historical dogma that proficiency in open thoracotomy was an absolute prerequisite for performing conventional VATS [54,55]. As long as this status quo persists, educating junior surgeons remains difficult until techniques are standardized and outcomes stabilize. While COVID-19 accelerated self-directed video-based learning, early-adopting institutions must continue to lead surgical education to convey crucial technical tips and pitfalls [56].

Regarding simulation training, RATS—exemplified by Intuitive Surgical’s *da Vinci* platform (Intuitive Surgical, Inc., Sunnyvale, CA, USA)—holds a distinct advantage over U-VATS due to its integrated video-based and virtual training modules, which offer educational capabilities unobtainable through traditional dry or wet labs [2,57,58,59]. Additionally, Intuitive Surgical actively drives the construction of collaborative networks bridging surgical societies and clinical centers. Supported by a robust socio-economic framework, this comprehensive ecosystem likely serves as a major catalyst for the rapid dissemination of RATS over U-VATS.

The broader dissemination of U-VATS faces notable educational barriers, particularly regarding the availability of structured mentorship. Unlike RATS—which benefits from a highly standardized, vendor-supported educational ecosystem including virtual reality simulation, structured proctoring, and credentialing curricula—U-VATS education has historically relied on individual surgical fellowships, observational visits, and initiative-driven workshops such as those led by JUVIG. Consequently, the scarcity of proficient U-VATS mentors remains a primary constraint.

Regarding institutional settings, these educational barriers are determined less by the rigid categorization of academic versus community hospitals and more by the presence of dedicated surgical leaders and institutional willingness to adopt uniportal techniques. In academic medical centers, structured training programs and hierarchical supervision often prioritize established M-VATS or RATS curricula, which may inadvertently slow the integration of U-VATS unless a department leader actively promotes it. Conversely, in community hospitals, the feasibility of U-VATS training hinges almost entirely on whether a proficient U-VATS surgeon is present; high-volume community centers with experienced mentors can achieve rapid, highly effective skill transfer, whereas facilities lacking such expertise face significant hurdles in initiating a program [60,61,62].

Furthermore, the relative paucity of prospective, long-term comparative evidence has historically made senior surgeons and decision-makers hesitant to shift established training paradigms. Upcoming prospective multicenter studies by JUVIG may help address this current evidence gap, which could in turn facilitate engagement with surgical leadership across both academic and community settings and support the establishment of standardized mentorship pathways.

Although uniportal VATS requires navigating instrument interference and restricted optics through a single incision while RATS involves multi-arm console control with 3-dimensional visualization, CUSUM-based evaluations indicate that both modalities share a comparable initial learning curve threshold—typically requiring 11 to 30 cases for uniportal VATS and approximately 20 cases for RATS—to achieve procedural competency in anatomical lung resections [7,30,63,64,65].

## 11. Evaluation Systems and Institutional Barriers

The Thoracoscopic Safety Technical Certification System, initiated by JACS (currently, JSTS) in 2021, aims to assess whether a surgeon can safely perform thoracoscopic lobectomy—primarily right upper lobectomy—rather than evaluating mere technical dexterity [66]. This initiative was established against the background of preventing fatal massive intraoperative hemorrhage caused by pulmonary vascular injury [67]. Any approach under the broad category of VATS, including M-VATS, U-VATS, and RATS, is eligible for application.

Although the evaluation criteria are uniform regardless of the surgical approach, a substantial disparity in pass rates was observed during the system’s inaugural year in 2021: 33.3% for U-VATS compared to 72.1% for M-VATS. By 2023, however, the pass rate for U-VATS improved markedly to 66.7%, while M-VATS reached 80.6% [67].

While these observed data demonstrate a clear initial gap and subsequent convergence, the underlying causes for the initial lower pass rate are likely multifaceted. Potential contributing factors on the candidate side include the early learning curve associated with U-VATS adoption, differences in applicant surgical experience, technical maturity, and familiarity with optimal video preparation for certification. Simultaneously, evaluation-related factors—such as the initial evolution and standardization of assessment criteria through repeated consensus meetings, alongside the reviewers’ early unfamiliarity with U-VATS-specific technical nuances (e.g., surgical field exposure, distal camera visualization, and non-grasping techniques) [68]—may have played a role. The subsequent rise in U-VATS pass rates by 2023 likely reflects a progressive refinement in surgical technique guided by evaluation feedback, enhanced video quality, increased standardization of grading metrics, and broader familiarity with uniportal techniques across the surgical community.

## 12. Global Perspectives and Future Outlook

**Figure 2:**
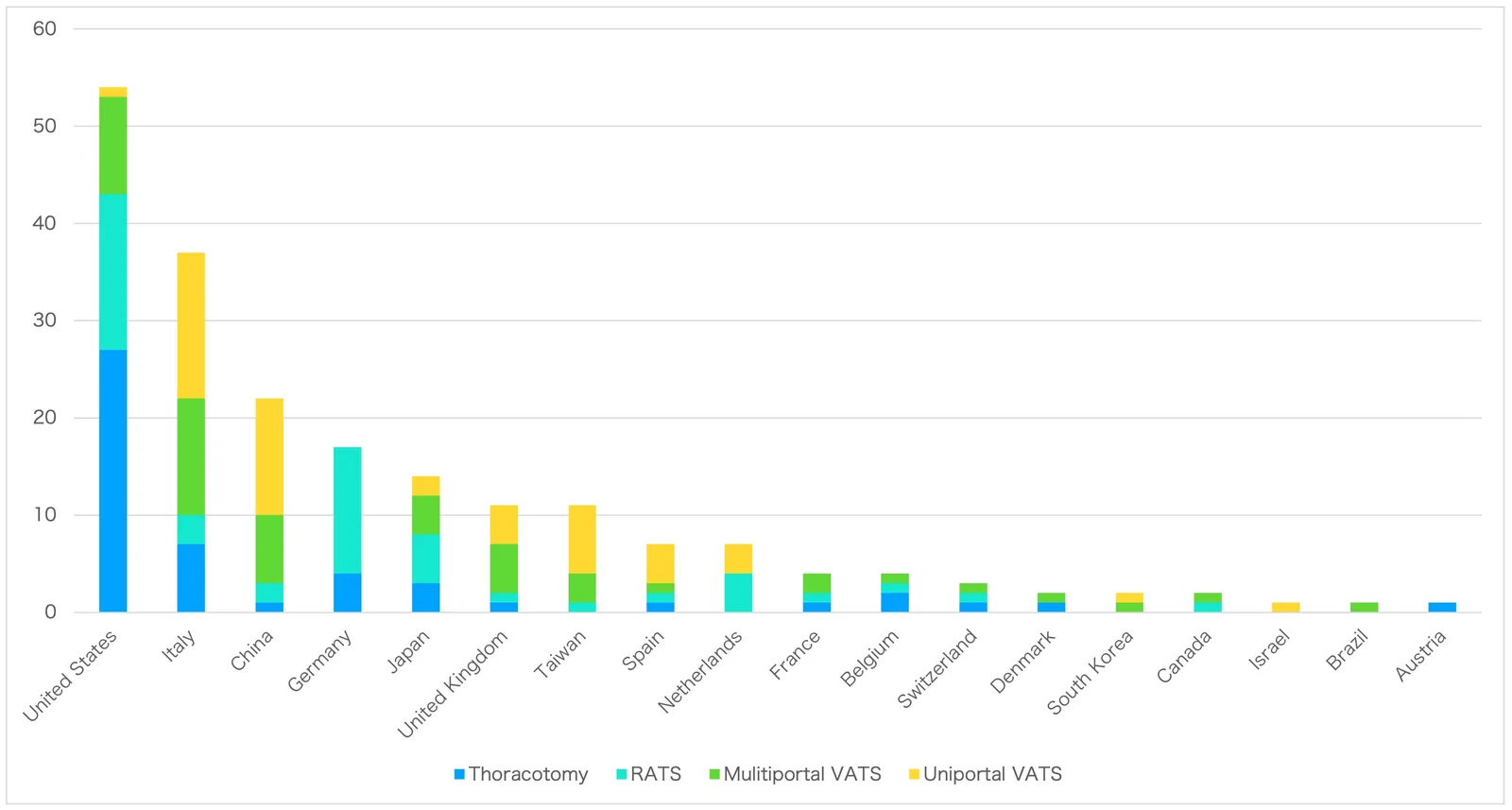
Geographic distribution of authors with high publishing volume identified in Dimensions.

The dissemination of U-VATS and RATS exhibits distinct geographic variation. These regional adoption patterns appear to be strongly influenced by key opinion leaders, the country of origin/development, and overall economic scale (Figure 2) [12,13,14,52,53,69,70]. Compared to RATS, U-VATS requires substantially lower initial capital expenditure and ongoing maintenance costs. Regarding robotic surgery, the market is no longer exclusive to Intuitive Surgical’s da Vinci platform, as numerous companies worldwide have entered the field and achieved clinical implementation [59,71,72]. In Japan alone, multiple indigenous platforms have emerged, including *hinotori* (Medicaroid Corporation, Kobe, Japan) [73] and *Saroa* (Riverfield Inc., Tokyo, Japan) [74]. Because these newer robotic systems generally offer lower initial acquisition and operational costs compared to the da Vinci system, RATS adoption is anticipated to accelerate further, expanding even into regions where robotic programs were previously cost-prohibitive.

Geographic distribution of the top 50 authors by publication volume (n = 50 per approach, total n = 200) across four surgical approaches: robot-assisted thoracic surgery (RATS), open thoracotomy, uniportal video-assisted thoracoscopic surgery (U-VATS), and multiportal VATS (M-VATS). Literature searches were conducted using Dimensions (Digital Science) to identify the top 50 authors for each approach, and the results were categorized and graphed by country.

## 13. Limitations

**Generalizability Beyond Lung Cancer Surgery**

A limitation of the present review is that the analyzed evidence is predominantly derived from anatomical lung resections for lung cancer, reflecting the current distribution of published literature. Nevertheless, many core principles—such as the pain-reducing benefits of single-port access in U-VATS, the technical advantages of robotic 3D visualization and wristed articulation in confined anatomical spaces (e.g., thymic and mediastinal tumor resections), and the institutional financial constraints under national reimbursement systems—are highly generalizable across broader thoracic surgical procedures. However, given the procedure-specific nuances and the relative paucity of high-level comparative data in esophageal, pediatric, and complex mediastinal surgeries, further dedicated studies are warranted to confirm modality-specific outcomes in these subspecialties.

**Distinction Between Academic Output and Clinical Practice**

Importantly, publication-based geographic productivity reflects academic research output and institutional infrastructure rather than nationwide clinical adoption rates or surgical expertise. Real-world adoption is largely shaped by healthcare economics, insurance coverage, and regulatory frameworks. Therefore, our bibliometric mapping should be interpreted strictly as an indicator of scholarly activity rather than direct clinical penetration or surgical skill.

## 14. Conclusions

Uniportal VATS represents a promising option for minimally invasive surgery. However, its widespread adoption for major and complex resections in Japan is restricted by both educational and societal barriers. Although RATS is expected to become increasingly widespread, VATS, U-VATS, and open thoracotomy will remain essential and maintain steady demand for the foreseeable future, driven by crucial educational, safety, and economic considerations.

## Figures and Tables

**Table 1:** Comprehensive comparison between uniportal VATS (U-VATS) and robot-assisted thoracic surgery (RATS).

Domain/Item	U-VATS	RATS
Surgical approach & port configuration	Single small incision (typically 3–4 cm).	Multiportal (typically 3–5 ports) or emerging single-port systems.
Surgical vision & visualization	2D or 3D thoracoscope, distal/angled visualization.	High-definition 3D binocular vision with motion scaling and tremor filtering.
Instrument dexterity & ergonomics	Rigid instruments, fulcrum effect; surgeon stands at bedside with potential physical fatigue.	Multi-articulated wristed instruments; seated ergonomic console.
Learning curve	Comparable overall learning curve; requires overcoming instrument interference and single-incision optical angles.	Comparable overall learning curve; allows intuitive console control due to wristed dexterity, though requiring bedside team coordination and docking proficiency.
Perioperative outcomes (morbidity & mortality)	Comparable to M-VATS and RATS in major pulmonary resections.	Comparable perioperative complication and mortality rates to M-VATS/U-VATS.
Postoperative pain & neuralgia	Potentially lower early pain and reduced intercostal nerve damage due to single incision.	Comparable or slightly higher intercostal nerve irritation due to multiple ports.
Lymph node dissection	Feasible; requires skilled non-grasping/exposure techniques in complex zones.	Excellent dexterity for deep mediastinal lymph node dissection without significant difference in long-term survival.
Conversion rate & emergency management	Low overall conversion rate; immediate bedside access allowing rapid extension of incision or conversion.	Low overall conversion rate; emergency conversion requires rapid undocking and clear bedside team readiness.
Oncological outcomes	Broadly comparable oncological adequacy and survival to M-VATS, with emerging/limited direct comparative data against RATS.	Broadly comparable oncological adequacy and survival to M-VATS, though direct comparative evidence with U-VATS remains limited.
Simulation & training infrastructure	Dependent on video-based learning, dry/wet labs, and direct operating room mentorship.	Comprehensive ecosystem with integrated virtual reality simulators and standardized curricula.
Capital & maintenance costs	Low initial capital investment; uses standard endoscopic instrumentation and lower consumables.	High initial capital cost and ongoing maintenance/disposable costs.

## Data Availability

The data used in this article will be shared upon reasonable request to the corresponding author, beginning 3 months and ending 5 years following article publication.
